# Summer Eating

**Published:** 1871-07

**Authors:** 


### SUMMER EATING.

We eat to keep warm and to sustain strength, and all articles of food have those two elements in varying proportion. Oils, tallow, and whale blubber are almost wholly of the warming element; hence in Greenland, where the thermometer is many degrees below zero, and a great deal of heat is required, a native will drink half a dozen gallons of oil every day, or eat ten pounds of tallow. In the hottest climates of the world the inhabitants live to a great extent on fruits and vegetables, which have but very little of the heating qualities. In our climate, which is between the two, meats, vegetables, and fruits are eaten all the year round; but if eaten judiciously, if eaten according to the season, — more of fruits and vegetables in summer, and less of meats and fats, — an incalculable amount of sickness would be prevented every year. We would think a man deranged who should keep as large fires burning in his house in summer as in winter, and yet we all persist in eating meats and fats and butter all through the summer. Meats and butter are on our tables three times a day, when in reality they ought to be sparingly used during the summer months, at least by the young, the old, and the feeble, and by all who are most of the time in-door, or who have no active employment. For the classes just named, a very appropriate diet for the summer would be as follows: —

Breakfast, — cold bread and butter, a slice of cold meat, or in its place a couple of eggs, or a saucer of berries or stewed fruit without milk, cream, or sugar. The same for dinner, with one vegetable; no other dessert. For supper, some cold bread and butter and a cup of hot drink, and nothing else; nothing whatever between meals. So far from starving on such a diet, the class of persons above named would thrive on it, would grow stronger every day, would have more bodily vigor, more mental elasticity, and a greater flow of animal spirits, and for the reason that few would eat too much; there would be nothing to overtempt the appetite, hence the stomach would not be overworked; what work it did perform, would be well done; the blood made would be pure, life giving, and energizing. Any man of ordinary intelligence and observation, who will give a fair trial to the above system of feeding, will scarcely fail to be convinced of its value within a week after he begins it.

